# Theta oscillations in 4-year-olds are sensitive to task engagement and task demands

**DOI:** 10.1038/s41598-019-42615-x

**Published:** 2019-04-15

**Authors:** Marlene Meyer, Hinke M. Endedijk, Freek van Ede, Sabine Hunnius

**Affiliations:** 1 Donders Institute for Brain Cognition and Behaviour, Radboud University, Nijmegen, The Netherlands; 2 Department of Psychology, University of Chicago, Chicago, USA; 3 Oxford Centre for Human Brain Activity, Wellcome Centre for Integrative Neuroimaging, Department of Psychiatry, University of Oxford, Oxford, UK; 4 Present Address: Montessorilaan 3, Nijmegen, 6525 HR The Netherlands

## Abstract

Top-down control processes are essential for guiding attention and working memory towards task-relevant information. Recently, theta oscillations were suggested as critical for these cognitive processes. Infant studies testing a mixture of bottom-up and top-down processes support adult theta findings. Yet, since infants cannot be instructed, it remains unclear to what extent theta oscillations are involved particularly in top-down control in early childhood. That is especially relevant towards school age when children need top-down control to solve the increasingly complex tasks. In this EEG study, we investigated whether theta-power in 4-year-olds is sensitive to task engagement and to different cognitive task demands. In a within-subjects design, children had three different instructions before watching videos including either no demands (No Task), language-related (Color-naming Task), or action-related (Imitation Task) demands. We analyzed children’s theta-power (3–6 Hz) in two contrasts: (1) Task vs. No Task and (2) Color-naming vs. Imitation Task. The findings revealed more frontomedial theta-power when children were engaged in a task and their frontomedial theta-power increased during their cognitive engagement. Theta-power was stronger over left fronto-temporal sites for language- compared to action-related demands. These findings support recent theoretical work highlighting theta oscillations in top-down control and extend this neurocognitive framework to preschoolers.

## Introduction

### Theta oscillations in adults: Top-down control processes

Theta oscillations in the brain and their potential functional role have fascinated cognitive neuroscientists for decades and motivated extensive research in the field of attention, memory and conflict monitoring. In human adults, the theta frequency range is referred to as the spectral band of 4–8 Hz^1^ with sources of theta being localized to particular brain regions. More precisely, theta oscillations recorded over frontal cortices (also called frontal theta) are associated with sources in medial frontal and anterior cingulate cortex (e.g.^1–3^), theta activity over such frontal and additional left temporal-parietal sites is associated with language processing^4,5^ and hippocampal theta likely reflects rhythmic activity of place cells in the hippocampus^6,7^. Until recently, the variety of cognitive processes that modulate theta oscillations left researchers wondering about their potential functional role. For instance, theta power is influenced by manipulations affecting attentional demands^8^, working memory load^9,10^, stimulus novelty^11^, and feedback and conflict monitoring^12^ as well as language processing^4^. Frontal theta power, in particular, is enhanced with increasing attentional demands of a task and recent MEG findings with adults suggest that theta coherence between frontal and hippocampal brain regions underlies complex forms of task-relevant memory integration^13^. Increase in frontal and left temporal theta power during retrieval of lexical-semantic information has been interpreted as topographically and potentially functionally distinct activation of nodes in the language processing network, like verbal working memory/syntactic processing (frontal theta) and retrieval of lexical meaning (left temporal)^4^. It is only recently that theoretical work and converging empirical evidence offer a new, possibly unifying perspective on the diverse findings on theta oscillations. It was proposed that frontal theta is involved in top-down control which guides the diverse cognitive processes^8,12^. Top-down control refers to cognitive control which based on prior knowledge guides a variety of processes towards task-relevant behavior. In contrast to bottom-up processes which strongly rely on external sensory input, top-down processes are based on prior knowledge alone and are “designed to enhance the neuronal processing of relevant sensory input […]”^14^^,(p. 148)^. As such, top-down control processes play an important role in guiding attention and working memory towards task-relevant information. Top-down control is thus not restricted to attentional processes alone but also guides processes like working memory, semantic retrieval, learning, error and feedback monitoring to adaptive behavior. Frontal theta oscillations are thought to exert top-down control over sensory processes through inter-areal communication with more posterior brain regions^15^. This is likely established through interaction with the phase and power of alpha and gamma oscillations^15^. Findings from adult electroencephalography (EEG) and magnetoencophalography (MEG) showing theta oscillations to be sensitive to task engagement, to the duration and the different demands of a task (for reviews see^8,12^; see also^4^) support the idea that theta oscillations are involved in task-relevant processing.

While we are beginning to understand the functional role of theta oscillations in adults, it is an open question whether top-down control is reflected in theta oscillations in young children. The brain regions that are implicated in the generation of frontal theta oscillations (such as medial frontal and anterior cingulate cortex) undergo large maturational changes in early childhood. When it comes to the functional role of theta oscillations in early childhood, however, there are only few studies to date. While outcomes of a small number of infant studies give first indications for a role of theta in infants’ sustained attention^16–18^, most developmental studies predominantly focused on bottom-up (i.e. stimulus driven) processing. As a consequence, the involvement of theta oscillations in top-down control processes, especially in solving externally imposed tasks early in life remains unclear. In particular, we investigated whether the involvement of frontal theta oscillations in task-relevant processing such as task engagement and different task demands is traceable in young children despite the immaturity of their medial frontal brain areas.

### Theta oscillations in early childhood: Bottom-up and top-down control processes

The general characteristics of theta oscillations in infants and young children, such as their spectral range and power, differ only marginally from those of adults. The spectral range of theta is slightly lower (around 3–6 Hz) and absolute theta power is typically higher in infants and young children than in older children or adults^6,19^. First exploratory studies on the functional role of theta in infants focused mainly on emotional processing^20,21^. Findings indicated higher theta power with higher emotional arousal both in association with positive and negative affect^21,22^. More recently, studies with infants and young children started to address the role of theta oscillations also for cognitive processing^19,23^. For instance, Orekhova and colleagues^19^ measured theta power while infants (aged 7- to 12-months) and young children (aged 4- to 6-years) explored toys and listened to child-directed speech, two situations which likely elicit a complex mixture of bottom-up capture of attention and top-down control. In both age groups, theta power increased during toy exploration and child-directed speech as compared to the baseline^19^. Similarly, Zhang and colleagues^24^ showed increased frontal theta in 6- to 12-month-old infants in reaction to exaggerated (i.e. infant-directed) compared to non-exaggerated speech. Together, these findings suggest that already early in life theta oscillations are likely modulated by stimulus-driven changes in attention potentially including aspects of top-down control. Besides this, recent developmental studies have investigated the involvement of frontal theta in memory processes^23^, speech perception^25^ and processing of social cues^26^. Although these studies provide first evidence for the role of theta oscillations in top-down control intertwined with bottom-up attentional processes, the evidence for involvement of frontal theta oscillations in uniquely top-down control early in life remains scarce to date.

### Theta oscillations in early childhood: Top-down control processes?

First evidence that theta oscillations might play a crucial role particularly in top-down control early on comes from a small number of infant studies investigating sustained attention^16–18^. Two of these studies made use of a peek-a-boo game during which a person hides behind an occluder before reappearing^16,17^. During the occlusion, infants were engaged in a period of sustained attention building up towards the reappearance of the person from behind the occluder. Comparable to findings by Stroganova and colleagues^17^ with 7- to 8-month-old infants, Orekhova and colleagues^16^ found increased theta power during the occluded period in 8- to 11-month-old infants. While the findings suggest that theta oscillations might be involved in top-down control early in life, infant studies like these are inherently limited by the constraint that tasks cannot be externally imposed through instruction. In this case the studies used contrasts between different stimuli (occlusion period vs. period during which a person was present) to investigate sustained attention. Whether the absence or presence of a task is reflected in modulations of theta oscillations without the stimulus confound remains an open question. To address this question, we are limited to ages in which young children are able to follow task instructions. Yet, research on the role of frontal theta in young children beyond their first birthday and especially in toddlers and preschoolers is extremely scarce. To our knowledge only three studies explored theta oscillations in young children during toddlerhood and preschool years^19,27,28^. The most recent study was conducted by Conejero and colleagues^27^ and showed increased frontal theta power for an unexpected (mismatching completion of puzzle) as opposed to an expected (matching) visual display in 16- to 18-month-old toddlers. These findings suggest the involvement of theta in error processing during toddlerhood. In the second study, Cuevas and colleagues^28^ presented 2-year-old toddlers with three items before removing them from sight. They were then asked to recall the previously presented items. Cuevas and colleagues found that the 2-year-olds’ theta power (3–5 Hz) was increased during recall of the items compared to baseline. In addition to these findings on memory, Orekhova and colleagues^19^ report attentional effects of theta in 4- to 6-year-old preschoolers as mentioned above. Here, theta power was higher during toy exploration and the presentation of child-directed speech as compared to a baseline.

Research on top-down control processes and the role of theta in preschoolers seems to be absent altogether. This is particularly surprising since the amount and variety of tasks children need to master increases considerably during early childhood, when children approach school age. Using top-down control processes to extract task-relevant information from the environment and to direct and maintain attention to task-relevant aspects is important for successful task performance. In adults, top-down control reflected in frontal theta power increases with prolonged attention to a task (see^8^ for a review) as well as working memory load^9^ and predicts behavioral benefits of attention and memory retrieval^6,8^. During early childhood significant structural and functional changes in frontal brain regions occur^29–31^. Yet, little is known about whether young children’s processing of task-relevant information is reflected in theta oscillations despite immaturity of the neural generators of theta oscillations.

### The current study

In the current study, we addressed this gap in knowledge by examining theta oscillations during top-down processing of task-relevant information in early childhood. More specifically, to examine whether the involvement of theta oscillations specifically in top-down control can be detected excluding bottom-up stimulus differences in young children, we investigated theta power changes in 4-year-old children in response to the engagement in and the specific cognitive demands of a task. To control for differences in bottom-up processing we kept the sensory input the same for the comparisons we performed. This experiment was part of a larger longitudinal study on young children’s social-cognitive development^32,33^ in which EEG was recorded in a subset of participants to investigate motor-related brain activity^34^. Although not originally designed to address this question, the current experimental set-up allowed us to look at the effects of task-related processing on theta power in a post-hoc analysis. While measuring their EEG, we presented 4-year-olds with short movie clips. Before watching the movie clips, children were instructed to either passively observe without a task (No Task), to subsequently name the color of a manipulated object in the scene (Color-naming Task) or to imitate the performed action they saw (Imitation Task). The movie clips, interleaved with a fixation cross, were presented for three consecutive times, before children had to respond or the next trial was initiated. The fixation cross stimuli were identical for all tasks. The movie clips in the Color-naming and Imitation Task were also identical but differed from the movie clips in the No Task condition.

To address our main research questions on involvement of theta oscillations in task engagement and different task demands, we compared whether theta power in young children differs between the presence and absence of a task (Color-naming & Imitation Task vs. No Task) and between different cognitive task-demands, requiring language-related (Color-naming Task) or motor-related (Imitation Task) processing. To exclude any potential bottom-up effects on theta oscillations, we contrasted theta power during identical stimulus periods only (i.e. the fixation cross period for the first contrast and movie clip period for the second contrast). If theta oscillations reflect top-down control processes early in life, then one would expect: (1) more theta power during task engagement than when not being engaged in a task, and (2) theta patterns to change with different cognitive task demands. While we did not have a directional hypothesis with respect to Color-naming and Imitation Task demands per se, we did hypothesize that different task demands would elicit different needs for top-down control – and possibly different areas involved in this control – and thus likely result in task-specific theta modulations. For instance, whereas the Color-naming Task affords auditory attention, lexical access and verbal rehearsal, the Imitation Task requires focused processing and encoding of the motor-related features. Since frontal theta was suggested to interact with attentional networks and memory formation depending on the current task demands (e.g., visual and auditory brain areas for visual and auditory attentional demands^8^), we expected theta to be sensitive to the language- and motor-related task demands in the current study, potentially reflected in language-related or motor-related brain areas.

In addition, the set-up of the study allowed us to investigate whether top-down control guiding sustained attention and encoding of information for later recognition would build up over time^8^. Previous findings in adults suggest that theta power increases with memory load and over time if attention needs to be sustained^8,10^. Thus, if frontal theta functions similarly in children, we hypothesized to find an increase of theta power over time (i.e., across the three consecutive stimulus presentations).

## Material and Methods

### Participants

Twenty-nine children (10 boys) aged 4 years (M = 52.48, SD = 1.94 months) participated in this EEG experiment. These participants came from a larger pool of children who took part in a longitudinal study on social-cognitive development (with a sample of over 100 children; see^32^). In this study, we only included a subset of participants who volunteered to participate in EEG research^34^. Children were accompanied to the testing session by their parents. Informed consent from a parent and/or legal guardian for study participation was obtained for all children. Participating families came from mixed socio-economic backgrounds and mostly from regions close to Nijmegen, a middle-sized urban city in the Netherlands. None of the children had indications of atypical development. For their participation, children received either a small gift (i.e. children’s book) or monetary compensation (20 euros). The study was carried out according to standard guides and regulations approved by the ethics committee at Radboud University Nijmegen.

### Procedure

To assess changes in oscillatory activity to task engagement and task demands, we recorded EEG in a within-subjects design with three conditions: No Task, a Color-naming Task and an Imitation Task. For this purpose, the children and their parents were invited to the EEG laboratory for a session of one hour. At the start of the session, children were familiarized with the experimenter and the equipment. They were fitted with a child-sized EEG cap (actiCap) with 32 active electrodes arranged in the 10–20 system with an online reference at FCz. We strived to keep all impedances below 60 kΩ when preparing the cap. Then, the experimental task was introduced in an adjacent electrically shielded test room.

**Figure 1:**
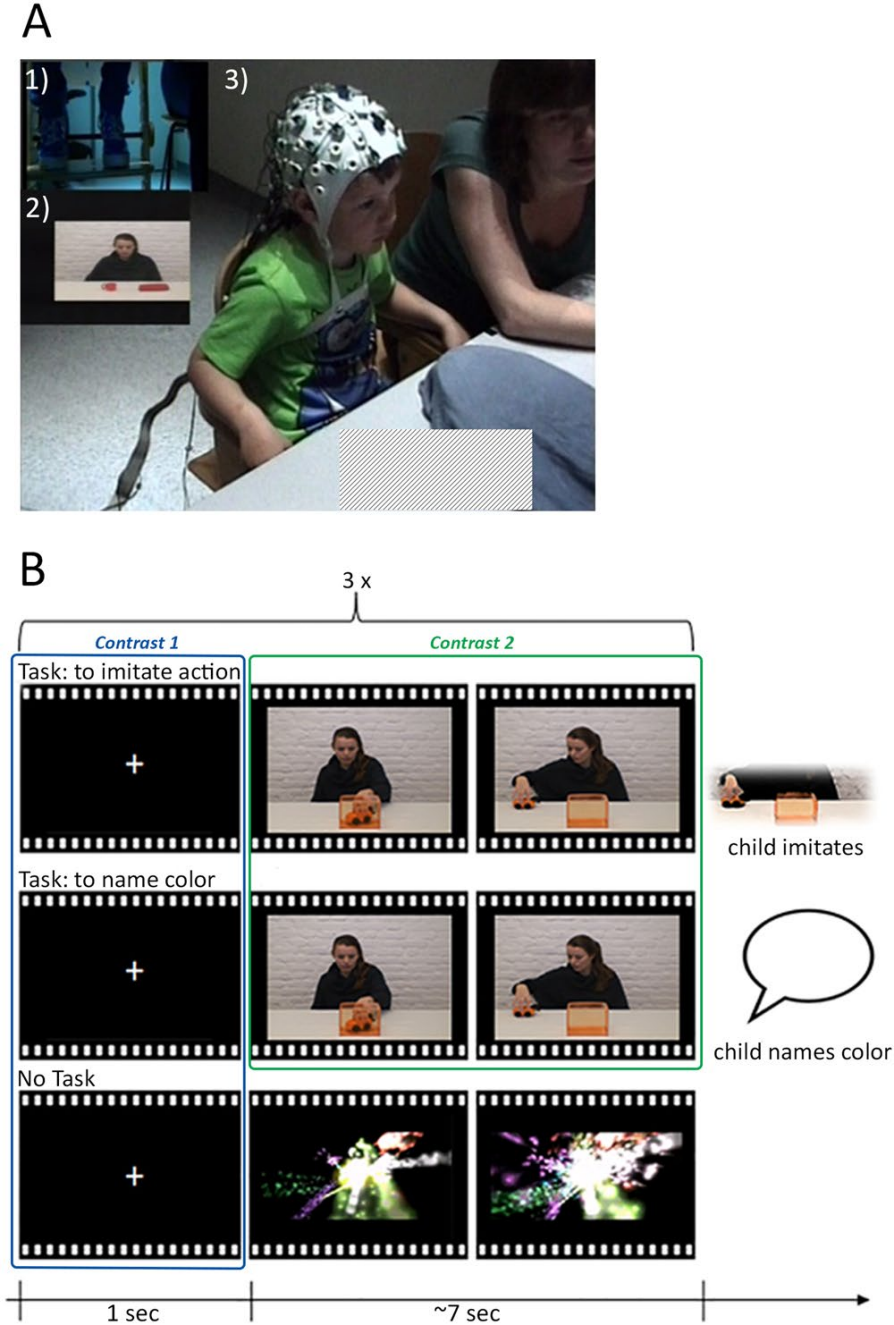
Panel A shows a still frame from the video recording of a 4-year-old child during the EEG recording. The video contains (1) a smaller video inlay showing the lower body of the child, (2) another inlay showing the stimulus display presented to the child and (3) the front view of the child. Panel B illustrates the experimental design including the three conditions (top row: Imitation Task; middle row: Color-naming Task, bottom row: No Task) and the corresponding visual display, as well as the contrasts used in the analysis (Contrast 1: Task vs. No Task; Contrast 2: Color-naming vs. Imitation Task). Note that each trial consisiting of a fixation period and movie clip was repeated three times before it was the child’s turn or the start of the next trial such that children saw the same movie clip three times in succession.

During the experimental task, the children were presented with two types of short movie clips. Movie clips displayed either goal-directed human actions (action clips) or abstract shape movements (non-human movement clips). There were six different action clips (e.g. moving a toy car in a box, stacking cups etc.) and six different non-human movement clips (similar to screensavers) each of which lasted approximately 7 seconds. The movie clips were presented for three consecutive times, each time interleaved with a fixation cross (for 1000 ms), before children had to respond or the next part was initiated. More specifically, the same movie clip was repeated three times before a response was required. Before watching the action clips, children were instructed to subsequently name the color of a manipulated object in the scene (Color-naming Task) or to imitate the action they observed (Imitation Task). The Color-naming and Imitation Tasks were presented in separate blocks, which were counterbalanced across children. Children thus saw each action in both tasks repeatedly. Moreover, before the non-human movement clips, children were instructed to passively watch without a task (No Task). The design is illustrated in Fig. 1 (note that informed consent for publication of identifying information/images for this Figure in online open-access publication has been granted). This design made it possible to address the question of whether or not (Task or No Task) children had to prepare for a language –related or action-related response (Color-naming or Imitation Task). Still, as this study is a re-analysis of a dataset collected to examine motor-related brain activity, we were confined to these custom-made stimuli in our analysis of theta oscillations. For this reason, we could not directly contrast task engagement between conditions for the movie periods but were limited to the fixation cross periods for this contrast (see Fig. 1B and EEG data analysis for details). Yet, most importantly, we kept the sensory input in all our task comparisons constant. This was accomplished by comparing identical movie periods to investigate different task demands (Color-naming Task vs. Imitation Task) and by contrasting children’s neural activity during presentation of the fixation cross to investigate theta oscillations during task engagement (Color-naming & Imitation Task vs. No Task; see Fig. 1B and EEG data analysis for more details).

Throughout the experimental task, children’s brain activity was recorded. More specifically, the signal was amplified using a BrainAmp DC EEG amplifier and digitized at 500 Hz with a band-pass filter of 0.1–125 Hz. Children were videotaped from two different angles that captured their upper and lower body (see Fig. 1A). These video recordings were coded offline to exclude time segments from the analysis in which children moved or did not look at the screen.

### EEG data analysis

The EEG data were analyzed with FieldTrip, an open source Matlab toolbox^35^. The data were segmented into events which were time-locked to the onset of the fixation cross and the movie clips. Data from movie clip events were additionally divided into 1-second segments to match the length of the fixation cross segments, thus resulting in seven 1-second segments (epochs) per movie clip. Segments were excluded from further analysis if children were moving or not looking at the stimulus during this period. The remaining segments were visually inspected for artifacts in the EEG to further remove noise due to eye-movement artifacts or noisy channels. One participant with no remaining trials for the No Task condition after artifact rejection was excluded from the final analysis. Cleaned data were re-referenced to the average of all electrodes, baseline corrected to the average of each segment and filtered with a DFT filter to remove line noise. Since frequency bands significantly shift throughout early childhood^36^, we first identified the sample-specific theta range for the current sample. For this purpose, we made use of the frequency bands previously identified for this specific sample of 4-year-old children based on a functional task^34^. Specifically, we used the lower alpha band boundary for this sample of children (i.e. at 7 Hz) to identify the upper boundary for theta at 6 Hz and to determine the theta band with a 3 Hz wide range to be 3–6 Hz. To determine power averaged over the theta band (3–6 Hz) we computed a fast Fourier transform using the multitaper method^37^ by centering the frequency of interest around 4.5 Hz with a 1.5 Hz frequency smoothing. For statistical comparisons of the conditions, we used cluster-based permutation tests^38^. For this, the power values were additionally normalized by means of a log transformation of each condition divided by the average of all conditions in the respective comparison. In the following, we separately describe the analyses of our main research questions (task engagement, task demands) and additional analyses (cognitive engagement building up over time). Note that for all comparisons we made sure that the sensory input was constant between conditions.

#### Analysis of theta power during task engagement (Task vs. No Task)

To address whether theta oscillatory activity in young children is sensitive to the general presence of a task, we compared children’s normalized theta power during observation of the fixation cross while they were engaged in a task (Task: Color-naming and Imitation Task pooled) compared to when they were not engaged in a task (No Task). For this comparison, we focused on the fixation cross periods because only in this period the sensory input was matched between the Task and No Task conditions (see Fig. 1B). Participants had on average 12 trials (range: 3–24) in the Task and 5 trials (range: 1–12) in the No Task condition. To test whether children had higher theta power while being engaged in a task than when not being engaged in a task, we used a cluster-based permutation test on the 3–6 Hz frequency bin of interest. Since these were post-hoc analyses which did not justify us to specify electrode sites a priori, we chose this procedure which includes all electrodes at test and accounts for multiple comparisons^38^. Thus, statistical testing was conducted on theta power over all electrode sites. To complement the findings descriptively and to be transparent about their spectral specificity, we additionally provide a 1 Hz-resolved frequency plot of potential clusters revealed by the test. However, to avoid biased analyses, no additional statistical test was conducted on the power values illustrated in these additional plots.

#### Analysis of theta power during different task demands (Color-naming vs. Imitation Task)

To examine changes in children’s theta activity to the different demands of a task we compared children’s processing of the action clips when they had the instruction to later name the color of the manipulated object in the scene (Color-naming Task) or to imitate the action they saw (Imitation Task). Thus, we compared normalized theta power values between the Color-naming and Imitation Task across all electrodes. For this comparison we focused on the EEG data acquired during the movie clip presentation for which sensory input was matched between both tasks (see Fig. 1B). On average participants had 56 trials (range: 19–130) for the Color-naming Task and 64 trials (range: 13–116) for the Imitation Task. Note that there are more trials in this comparison than the task engagement comparison because the longer movie periods contained more data segments than the fixation cross stimuli. We again used a cluster-based permutation test to contrast the two conditions within the 3–6 Hz frequency range. Finally, to explore the specificity of any findings with respect to the theta frequency range, we provide frequency plots illustrating power in higher and lower frequencies (2–14 Hz). Analogous to the theta power analysis during task engagement, no statistical testing was conducted for the additional frequency plots as they merely serve descriptive and transparency purposes (as suggested by^39^). See Supplementary Material for a complementary analysis on the fixation cross period which is not included in the main manuscript due to limited amount of data for this contrast. For transparency, the supplementary material also provides additional analyses on the alpha band for the task engagement and task demands contrast (for results see Supplementary Figs 2 and 3).

#### Analysis of theta power unfolding with prolonged task engagement (Task)

In addition, we tested whether theta power would build up over time the longer children were required to focus during a task. For this purpose, we compared children’s theta power across the first, second and third presentation of the fixation cross within a task block (Color-naming and Imitation Task). (Note that there were not enough data to split either the No Task or the individual tasks (Color-naming and Imitation Task) into the three order presentations. These comparisons can thus not be provided in this study.) During the presentation of the stimuli, the fixation cross preceded each action clip. Therefore, the presentation of the first fixation cross preceded any information children had to make use of for their task (i.e. detecting and remembering the color or encoding to later imitate the action). However, during the second and third presentation of the fixation cross children needed to sustain their attention, encode the information of the movie clips for later use and prepare as well as inhibit their response. To test whether theta power reflected this build up over time, we contrasted the power values across presentation order (i.e. first, second, third) using a within-subjects test. Power values were pooled over electrode sites which were found significantly different between conditions (Task vs. No Task) in the main comparison. Eleven participants who lacked data of either presentation order were excluded from this comparison. The remaining eighteen participants had on average 2 trials (first), 5 trials (second) and 6 trials (third) left for the comparison (ranges: 1–7, first; 1–10, second; 2–9, third). Although the number of trials for this comparison is relatively low, it can nevertheless provide important information that add to our analysis of the functional role of theta in young children. Since the data violated the assumptions of the repeated ANOVA, we used a non-parametric test (i.e. Friedman Test).

## Results

### Task performance

Overall, the 4-year-old children could successfully perform both tasks: All children imitated all actions in the Imitation Task correctly. Twenty-six children named all colors correctly, two children named one out of six colors incorrectly and one child named three out of six colors incorrectly.

### Theta power during task engagement

**Figure 2:**
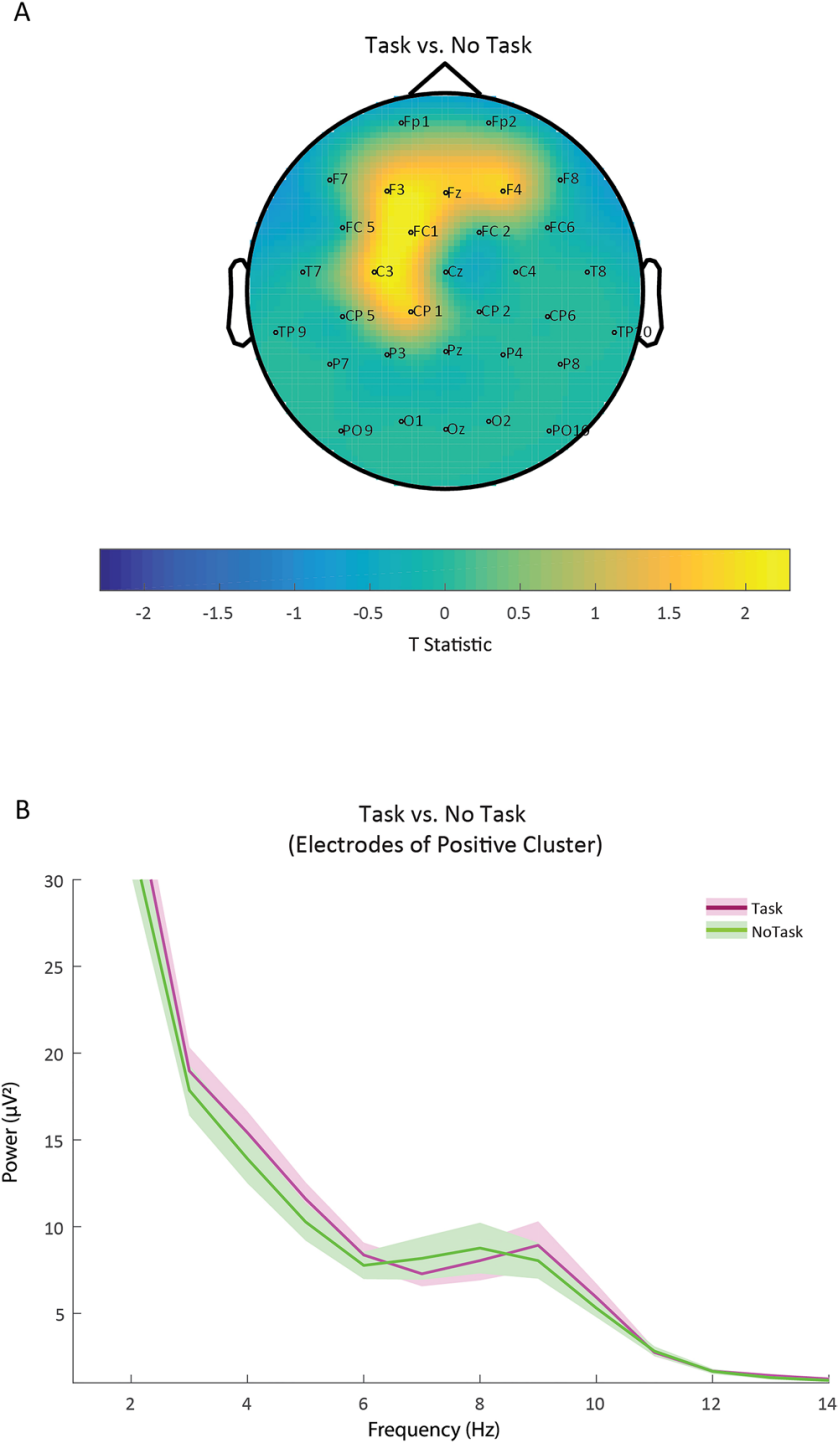
Panel A illustrates the topography of the results as determined by the cluster-based permutation test contrasting the Task and No Task conditions. As indicated by the warmer colors on the spectrum (reflecting the t statistic, with critical t values at +/−2.052), the difference was most pronounced at the fronto-central electrode sites. That is, theta power (3–6 Hz) measured at these sites (F3, Fz, F4, FC1, C3, CP1) was higher in the Task than in the No Task condition. Note that this comparison is based on the fixation cross period. Panel B displays power values as a function of frequency (Hz) separately for the Task (pink) and No Task (green) condition. Power values were averaged across electrode sites of the positive cluster (F3, Fz, F4, FC1, C3, CP1). Shaded areas represent the standard error of power values.

Comparing theta power (3–6 Hz) when children were engaged in a task to when they did not have a task (by means of a cluster-based permutation test) yielded a significant difference (*p* < 0.05) which was pronounced in six adjacent fronto-central electrodes (F3, Fz, F4, FC1, C3, CP1). Figure 2A illustrates the topography of this effect. Normalized theta power values over these fronto-central sites were significantly higher in the presence of a task than when children did not have a task. We descriptively explored whether this effect held for other frequency ranges as well or whether it was specific to the theta frequency range. For this purpose, we computed a 1 Hz-resolved frequency plot of the positive cluster found by the cluster-based permutation test. The frequency plot is displayed in Fig. 2B. Figure 2B suggests that in contrast to higher frequencies (i.e. Task < No Task in 6–8 Hz) a constant power difference was observed throughout children’s theta frequency band of 3 to 6 Hz.

### Theta power during different task demands

**Figure 3:**
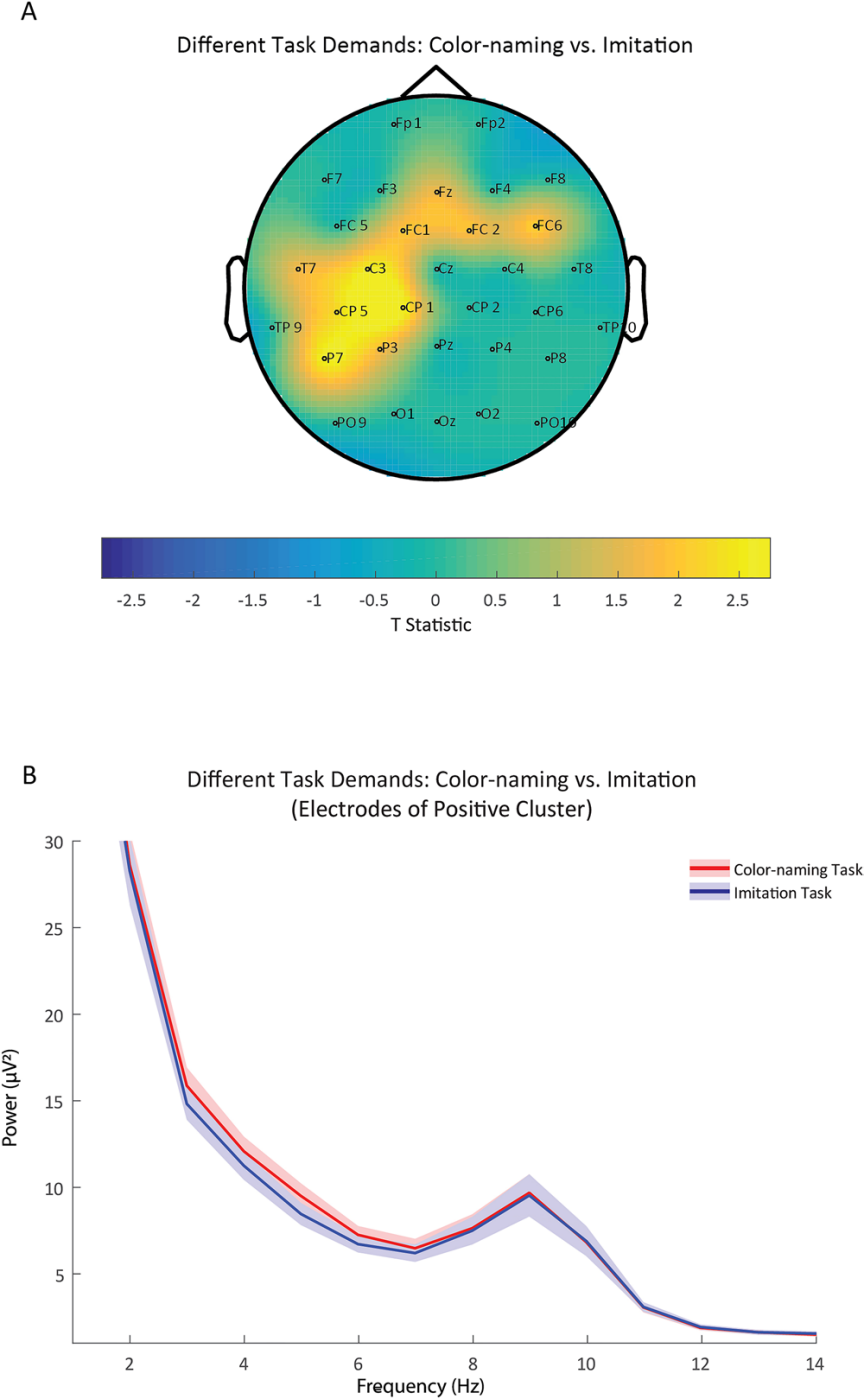
Panel A illustrates the topography of the results as determined by the cluster-based permutation test contrasting the Color-naming and Imitation Task conditions. As indicated by the warmer colors on the spectrum (reflecting the t statistic, with critical t values at +/−2.052), the difference was most pronounced at the fronto-temporal electrode sites. The topography shows the effect to be predominantly left-lateralized. That is, theta power (3–6 Hz) measured at these sites (Fz, FC1, FC2, FC6, T7, C3, CP5, CP1, P7, P3) was significantly higher in the Color-naming than in the Imitation Task condition. Note that this comparison is based on the action clip period. Panel B displays power values as a function of frequency (Hz) separately for the Color-naming (red) and Imitation Task (blue) condition. Power values are averaged across electrode sites of the positive cluster (Fz, FC1, FC2, FC6, T7, C3, CP5, CP1, P7, P3). Shaded areas represent the standard error of power values.

We investigated children’s theta activity to different task demands (i.e. to later name a color or imitate an action) by means of a cluster-based permutation test. As illustrated in Fig. 3A, our analyses yielded a significant difference (p < 0.05) which was pronounced in ten, mostly left-lateralized, fronto-temporal electrodes (Fz, FC1, FC2, FC6, T7, C3, CP5, CP1, P7, P3). Normalized theta power values were significantly higher in the Color-naming Task compared to the Imitation Task. Further descriptive examination of this effect across other frequency bands suggests that it was specific to the theta frequency range typically observed in young children and involved the same range in which the influence of task presence was apparent (see Fig. 3B, cf Fig. 2B). It is noteworthy that, in this comparison, the specificity to the theta frequency range is more apparent than for the task engagement comparison discussed above. This might reflect that in addition to theta also other rhythms (see Supplementary Material for additional analyses on the alpha band) are involved in task engagement, while the current task demands specifically involved theta activity. Another possibility is that the higher signal-to-noise ratio in the task demand contrast allows for a clearer dissociation between frequency bands. A definitive conclusion as to what is underlying this pattern cannot be drawn based on the current results.

### Theta power unfolding with prolonged task engagement

**Figure 4:**
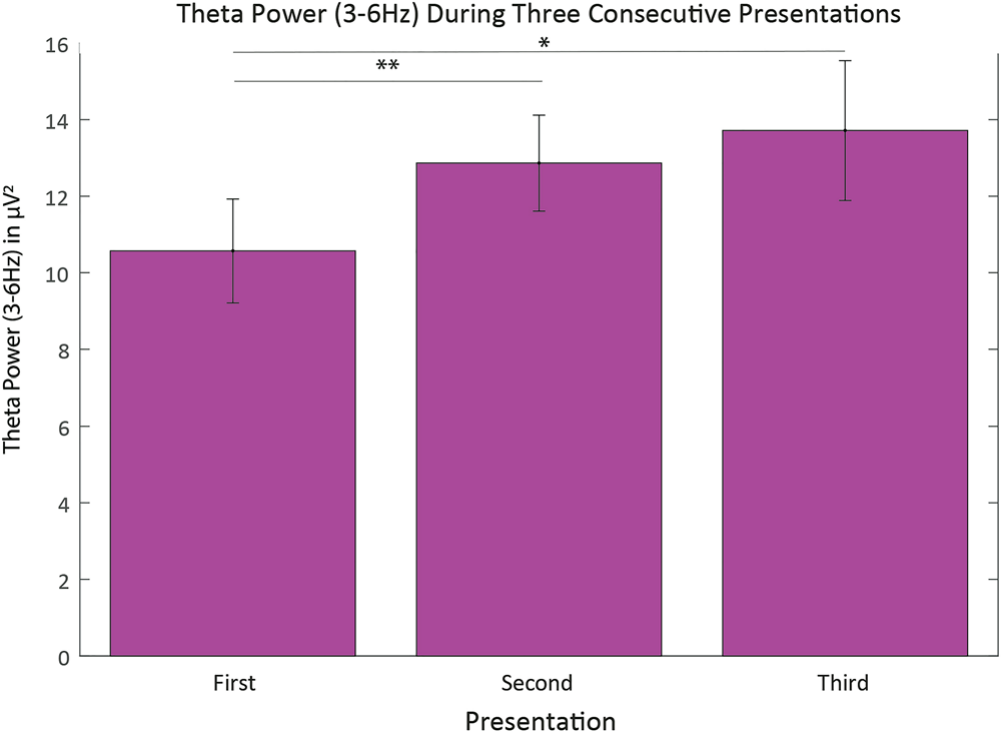
Bar graph representation of theta power (3–6 Hz) in the first, second, and third presentation of the fixation cross during the task block (i.e. Color-naming and Imitation Task), with *indicating p < 0.05 and **indicating p < 0.025. Note that this comparison is based on the fixation cross period and includes only the subset of participants who contributed data to all of the three presentations. Vertical error bars represent the standard error.

During the task blocks, before children had to respond they observed three consecutive presentations of a movie clip preceded by a fixation cross. To investigate whether theta power builds up across these three sequential presentations we compared the three fixation cross periods. Children’s fronto-central theta power was significantly affected by the consecutive presentation, reflecting changes over time (χ^2^(2) = 7.0, *p* < 0.05), as illustrated in Fig. 4. We followed up on this effect by means of Wilcoxon tests comparing the first with the second and second with third presentation. Bonferroni correction was applied to correct for multiple tests (significance level at *p* < 0.025). Theta power increased significantly between the first and second presentation of a stimulus (*T* = 32, *r* = −0.39). Theta power during the second and third presentation, however, did not significantly differ from each other despite a descriptive trend (*T* = 79, *ns*, *r* = −0.05). Thus, children’s theta power over fronto-central regions increased significantly after they first had to encode information, sustain attention and prepare as well as inhibit a response following the first action clip (i.e. presented between the first and second fixation cross).

## Discussion

### Theta oscillations in early childhood: Top-down control processes

We set out to investigate the functional role of theta band activity in early childhood. Recent theoretical approaches have united a multitude of empirical findings on theta reactivity in adults by suggesting theta to be a driving force in top-down control processes which guide attention and working memory^8,12^. In a post-hoc reanalysis of a unique dataset of 4-year-old children collected with EEG^34^ we tested whether theta power (3–6 Hz) in 4-year-old children is sensitive to top-down control that is required when being engaged in a task while excluding bottom-up capture of attention. Our results show that children had higher theta power over fronto-central brain areas when they were engaged in a task than when they were not involved in a task. This change in theta power and the frontal topography of this effect are consistent with adult findings of frontal theta^6,8,12^. Examination of other frequency ranges supports the specificity of this effect to the theta band in young children. Notably, this effect was observed while children had identical sensory input (i.e. observing a fixation cross on the screen) accounting for bottom-up processing. These findings therefore indicate that already at this young age when medial frontal brain regions are not yet fully matured, children’s frontal theta activity is sensitive to cognitive task engagement which requires top-down control.

In addition, we tested whether children’s theta power was affected by the different cognitive demands of a task. While their brain activity was being measured, children were engaged in a language-related and a motor-related task. While we did not have a directional hypothesis, we found that children had higher theta power over left fronto-temporal electrode sites when being engaged in the language-related task compared to the motor-related task. The lateralization of this topography to the left hemisphere is consistent with previous adult findings on theta oscillations during language-related processing which requires lexical activation and verbal rehearsal^4,5^. Interestingly, like in the adult findings^4^ both, frontal and left temporal sites, showed increased theta power. This topographic distribution could be reflective of two distinct or one overarching function of theta oscillations in processing specific task demands in early childhood. Two distinct functions might be at play in that frontal theta oscillations exert top-down control over retrieval of word meaning which in turn is reflected in left temporal theta activation. Thus, in addition to frontal theta that may reflect domain-general top-down cognitive control, there may also be complementary theta sources that reflect, function-specific control, such as linguistic access. This is in line with the notion of inter-areal communication between frontal theta band oscillations and oscillatory power associated with various cognitive functions in other brain areas^8,12^. Alternatively, the distributed theta activation might reflect one function, namely top-down control of theta oscillations spanning over both frontal and left temporal regions. As in the previous adult studies on language processing^4,5^ the precise functional nature of this topographic distribution remains speculative. Still, the findings clearly suggest that theta oscillations in young children are sensitive to specific cognitive task demands. As for task engagement, the descriptive spectral pattern suggests that this effect was specific to the theta frequency range. It is thus not only the engagement in a task but also the different cognitive demands of a task that affect theta oscillations in young children.

Top-down control processes are also important for sustaining attention to and encoding information of task-relevant stimuli as well as maintaining response preparation over a prolonged period of time. We therefore investigated to what extent prolonged task engagement would be reflected in children’s frontal theta activity. In this experiment, children observed three sequential presentations of fixation crosses followed by movie clips before they had to respond. As a consequence, during the task engagement blocks, the first fixation period only engaged preparatory cognitive control. In contrast, the second and third fixation periods likely engaged retrospective cognitive control processes (e.g. sustaining attention and keeping task-relevant information like color and action in mind) as well as response preparations and its inhibition in addition. If theta power scales with the cognitive control processes involved, then one would expect more theta power during the second and third fixation periods. This is precisely what we observed. Our findings indeed show that children’s frontal theta power increased significantly during task engagement after they have received task-relevant information but before they could make use of it in their overt response. This is in agreement with adult findings of theta power increasing over time with longer task-involvement^40^, and higher attentional^8^, and memory load^9^. Although the same movie clip was repeated three times, imposing potentially lower memory load than three distinct movie clips would have, children still needed to hold the observed information in mind until acting upon it. In interesting direction for future research would be to systematically manipulate the degree of top-down control.

The current findings are based on post-hoc analyses that were limited by the existing experimental design. For instance, we were confined to the fixation cross period in the task engagement comparison to ensure identical sensory input between conditions. The results on task engagement might therefore to some extent reflect task performance monitoring and task preparation ahead of the upcoming movie stimulus in addition to and as part of task engagement. Also the amount of trials in the analysis was limited, a known constraint of developmental EEG research^41^. Therefore, the interpretation of the results should be considered with some caution. Still, the current findings are based on careful statistical comparisons controlling for the false-alarm rate and provide clear indications of the functional role of theta in top-down control in early childhood. All in all, the outcomes complement infancy research on theta reactivity during top-down and bottom-up attention^16,17,19^ by offering evidence for neural underpinnings of top-down control needed to solve externally imposed tasks in early childhood. In this period in which young children approach school age, they need to gradually learn how to master the various cognitive task demands they are confronted with. Previous findings have associated theta power in adults with their performance on a task. For instance, when adults were asked to later recall items from memory, they were significantly better in the recall when their theta synchronization was larger during encoding of the relevant information^6^. At an older age (i.e. around 60–70 years), adults show a decline in memory performance which is accompanied by a decrease in frontal theta power^42^. Thus, cognitive task performance seems tightly linked to frontal theta reactivity. In the current task, children performed at ceiling level such that we could not investigate the relation between theta activity and children’s performance. Yet, the degree to which children engage in top-down control, as reflected in theta activity, might predict their behavioral success in a more difficult task. Neurofeedback studies suggest that older children can successfully train to change their level of frontomedial theta power^43^. In children with autism spectrum disorder (ASD) this was associated with an improvement in executive functioning^43^. These findings suggest that a potential malfunctioning of the anterior cingulate cortex, one of the generators of theta oscillations, can be influenced by neurofeedback. Examining the relation between theta activity and behavioral performance and manipulating theta levels is a promising avenue for future research with potential for insights in early executive functioning development and atypical development.

## Conclusions

The current findings suggest that top-down control required by task engagement modulates frontomedial theta power in young children. Prolonged cognitive engagement during a task increases children’s theta power gradually, and different task demands additionally modulate theta power. More specifically, language-related demands (such as retrieving a color-word) were reflected in theta modulations over left-lateralized fronto-temporal brain regions in 4-year-olds. Our findings provide the basis for using theta oscillations as a neural marker of top-down control in developmental research investigating early childhood. As such, theta oscillations have the potential to provide insight into aspects of early development that are not directly reflected in overt behavior but traceable in neural processes. For instance, processes guided by top-down control such as working memory, sustained attention and inhibition could be examined using theta oscillations as neural marker early in life. For cognitive neuroscientists, these findings raise the question as to which role exactly the medial frontal and anterior cingulate cortex play in the generation and function of frontal theta. Although these areas still undergo significant structural and functional changes during early childhood, frontal theta clearly reflects top-down control processes. It is thus unclear which minimal structural and functional requirements these areas need to fulfill for the generation of functional frontal theta oscillations for top-down-control. Future research is needed to address this question. To sum up, our findings support recent theoretical work that highlights the role of theta oscillations in top-down control and provide evidence for the sensitivity of theta activity to the engagement in and different demands of a task in young children.

## Supplementary information

Permission for Figure 1

Supplementary Analyses

## Data Availability

The dataset generated during the current study and the results obtained through analysis of this dataset is available in the Donders Research Data repository, http://hdl.handle.net/11633/di.dcc.DSC_2018.00119_275. Prior to accessing and downloading the shared data, users must create an account. It is possible to use an institutional account, an ORCID account, or a social ID from Google+, Facebook, Twitter, LinkedIn or Microsoft. After authentication, users must accept the Data Use Agreement (DUA), after which they are automatically authorised to download the shared data. The DUA specifies whether there are any restrictions on how the data may be used. Instructions for how to request access and to download shared data can be found at http://www.ru.nl/donders/research/data/user-manual/access-shared-data/. The Radboud University and the Donders Institute for Brain, Cognition and Behaviour will keep these shared data available for at least 10 years. The video recordings generated during the current study are not publicly available due to privacy reasons (i.e. data are identifiable).

## References

[1] Hsieh LT, Ranganath C (2014). Frontal midline theta oscillations during working memory maintenance and episodic encoding and retrieval. Neuroimage.

[2] Ishii R (1999). Medial prefrontal cortex generates frontal midline theta rhythm. Neuroreport.

[3] Meltzer JA, Negishi M, Mayes LC, Constable RT (2007). Individual differences in EEG theta and alpha dynamics during working memory correlate with fMRI responses across subjects. Clinical Neurophysiology.

[4] Bastiaansen M, Linden MVD, Keurs MT, Dijkstra T, Hagoort P (2005). Theta responses are involved in lexical—Semantic retrieval during language processing. Journal of Cognitive Neuroscience.

[5] Piai V, Roelofs A, Jensen O, Schoffelen JM, Bonnefond M (2014). Distinct patterns of brain activity characterise lexical activation and competition in spoken word production. PloS one.

[6] Klimesch W (1999). EEG alpha and theta oscillations reflect cognitive and memory performance: a review and analysis. Brain research reviews.

[7] O’Keefe J, Recce ML (1993). Phase relationship between hippocampal place units and the EEG theta rhythm. Hippocampus.

[8] Clayton MS, Yeung N, Kadosh RC (2015). The roles of cortical oscillations in sustained attention. Trends in Cognitive Sciences.

[9] Jensen O, Tesche CD (2002). Frontal theta activity in humans increases with memory load in a working memory task. European Journal of Neuroscience.

[10] van Ede Freek, Jensen Ole, Maris Eric (2017). Supramodal Theta, Gamma, and Sustained Fields Predict Modality-specific Modulations of Alpha and Beta Oscillations during Visual and Tactile Working Memory. Journal of Cognitive Neuroscience.

[11] Cavanagh JF, Zambrano‐Vazquez L, Allen JJ (2012). Theta lingua franca: A common mid‐frontal substrate for action monitoring processes. Psychophysiology.

[12] Cavanagh JF, Frank MJ (2014). Frontal theta as a mechanism for cognitive control. Trends in Cognitive Sciences.

[13] Backus AR, Schoffelen JM, Szebényi S, Hanslmayr S, Doeller CF (2016). Hippocampal-prefrontal theta oscillations support memory integration. Current Biology.

[14] Sarter M, Givens B, Bruno JP (2001). The cognitive neuroscience of sustained attention: where top-down meets bottom-up. Brain research reviews.

[15] Voytek, B. *et al* Oscillatory dynamics coordinating human frontal networks in support of goal maintenance. *Nature Neuroscience* (2015). 10.1038/nn.4071 PMC4551604 26214371

[16] Orekhova EV, Stroganova TA, Posikera IN (1999). Theta synchronization during sustained anticipatory attention in infants over the second half of the first year of life. International Journal of Psychophysiology.

[17] Stroganova TA, Orekhova EV, Posikera IN (1998). Externally and internally controlled attention in infants: an EEG study. International Journal of Psychophysiology.

[18] Xie, W., Mallin, B. M. & Richards, J. E. Development of infant sustained attention and its relation to EEG oscillations: an EEG and cortical source analysis study. *Developmental Science*, 1467–7687 (2017). 10.1111/desc.12562 PMC5628078 28382759

[19] Orekhova EV, Stroganova TA, Posikera IN, Elam M (2006). EEG theta rhythm in infants and preschool children. Clinical Neurophysiology.

[20] Kugler J, Laub M (1971). Puppet show theta rhythm. In Electroencephalography and Clinical Neurophysiology.

[21] Maulsby RL (1971). An illustration of emotionally evoked theta rhythm in infancy: Hedonic hypersynchrony. Electroencephalography and Clinical Neurophysiology.

[22] Futagi Y, Ishihara T, Tsuda K, Suzuki Y, Goto M (1998). Theta rhythms associated with sucking, crying, gazing and handling in infants. Electroencephalogr Clin Neurophysiol.

[23] Begus K, Southgate V, Gliga T (2015). Neural mechanisms of infant learning: differences in frontal theta activity during object exploration modulate subsequent object recognition. Biology Letters.

[24] Zhang Y (2011). Neural coding of formant‐exaggerated speech in the infant brain. Developmental Science.

[25] Bosseler, A. N. *et al*. Theta brain rhythms index perceptual narrowing in infant speech perception (2013). 10.3389/fpsyg.2013.00690 PMC3795304 24130536

[26] Michel C (2015). Theta-and alpha-band EEG activity in response to eye gaze cues in early infancy. NeuroImage.

[27] Conejero Ángela, Guerra Sonia, Abundis-Gutiérrez Alicia, Rueda M. Rosario (2016). Frontal theta activation associated with error detection in toddlers: influence of familial socioeconomic status. Developmental Science.

[28] Cuevas K, Raj V, Bell MA (2012). A frequency band analysis of two-year-olds’ memory processes. International Journal of Psychophysiology.

[29] Casey BJ, Giedd JN, Thomas KM (2000). Structural and functional brain development and its relation to cognitive development. Biological Psychology.

[30] Giedd JN (1999). Brain development during childhood and adolescence: a longitudinal MRI study. Nature Neuroscience.

[31] Le Magueresse C, Monyer H (2013). GABAergic interneurons shape the functional maturation of the cortex. Neuron.

[32] Endedijk HM, Cillessen AH, Cox RF, Bekkering H, Hunnius S (2015). The Role of Child Characteristics and Peer Experiences in the Development of Peer Cooperation. Social Development.

[33] Endedijk HM (2015). Development of interpersonal coordination between peers during a drumming task. Developmental Psychology.

[34] Endedijk H, Meyer M, Bekkering H, Cillessen A, Hunnius S (2017). Neural Mirroring and Social Interaction: Motor System Involvement During Action Observation Relates to Early Peer Cooperation. Developmental Cognitive Neuroscience.

[35] Oostenveld, R., Fries, P., Maris, E. & Schoffelen, J. M. FieldTrip: open source software for advanced analysis of MEG, EEG, and invasive electrophysiological data. *Computational Ïntelligence and Neuroscience*, 2011 (2011). 10.1155/2011/156869 PMC3021840 21253357

[36] Berchicci M (2011). Development of mu rhythm in infants and preschool children. Developmental Neuroscience.

[37] Percival, D. B. & Walden, A. T. *Spectral analysis for physical applications*. Cambridge University Press (1993).

[38] Maris E, Oostenveld R (2007). Nonparametric statistical testing of EEG-and MEG-data. Journal of neuroscience methods.

[39] van Ede F, Maris E (2016). Physiological plausibility can increase reproducibility in cognitive neuroscience. Trends in Cognitive Sciences.

[40] Wascher E (2014). Frontal theta activity reflects distinct aspects of mental fatigue. Biological Psychology.

[41] Stets M, Stahl D, Reid VM (2012). A meta-analysis investigating factors underlying attrition rates in infant ERP studies. Developmental Neuropsychology.

[42] Kardos Z, Tóth B, Boha R, File B, Molnár M (2014). Age-related changes of frontal-midline theta is predictive of efficient memory maintenance. Neuroscience.

[43] Kouijzer ME, de Moor JM, Gerrits BJ, Congedo M, van Schie HT (2009). Neurofeedback improves executive functioning in children with autism spectrum disorders. Research in Autism Spectrum Disorders.

