# Supplementary material for: Theta oscillations in 4-year-olds are sensitive to task engagement and task demands
Source: Sci Rep. 2019 Apr 15;9:6049. doi: 10.1038/s41598-019-42615-x (PMC6465288; doi:10.1038/s41598-019-42615-x)
Supplement: Supplementary file 1 — Permission for Figure 1 [file 41598_2019_42615_MOESM1_ESM.pdf]

Donders Institute
for Brain, Cognition and Behaviour

Centre for Cognition
Montessorilaan 3
6500 HE Nijmegen
The Netherlands

November, 2018

To whom it may concern,

Permission is granted to (Springer Nature Limited), to publish the image (Figure 1) under a CC BY open access license. Permission is granted to publish the image in all formats i.e. print and digital.

Yours sincerely,
Marlene Meyer & Hinke M. Endedijk (the woman on the picture)
