# Supplementary material for: Theta oscillations in 4-year-olds are sensitive to task engagement and task demands
Source: Sci Rep. 2019 Apr 15;9:6049. doi: 10.1038/s41598-019-42615-x (PMC6465288; doi:10.1038/s41598-019-42615-x)
Supplement: Supplementary file 2 — Supplementary Analyses [file 41598_2019_42615_MOESM2_ESM.docx]

**Supplementary Material:**

**Theta oscillations in 4-year-olds are sensitive to task engagement and task demands**

**M. Meyer^1,2*^, H. M. Endedijk^1^, F. van Ede^1,3^, S. Hunnius^1^**

**^1^**Donders Institute for Brain, Cognition and Behaviour, Radboud University Nijmegen, The Netherlands

**^2^**Department of Psychology, University of Chicago, USA

**^3^** Oxford Centre for Human Brain Activity, Wellcome Centre for Integrative Neuroimaging, Department of Psychiatry, University of Oxford, Oxford, UK

***^*^Corresponding Author (Present Address)***

m.meyer@donders.ru.nl

Montessorilaan 3
6525 HR Nijmegen

The Netherlands

***Additional analysis:***

**Theta power during different task demands during fixation cross period**

For this analysis, theta power during the fixation cross period were compared between the Color-Naming and Imitation Task. Two additional participants had to be excluded from this contrast because they did not provide at least 1 trial for each condition. On average participants had 6 trials (range: 1-14) for the Color-naming Task and 6 trials (range: 1-12) for the Imitation Task for this contrast. Analogous to the main analyses, we used a cluster-based permutation test to contrast the two conditions within the 3-6Hz frequency range. Finally, to explore the spectral distribution, we provide a frequency plot illustrating power in frequencies between 2-14Hz for both conditions. The cluster-based permutation test of this contrast did not reveal any positive clusters. The topographic distribution of the contrast is shown on the left side of panel A, indicating the normalized power difference between conditions. The topographic plot on the right in panel A indicates that no positive cluster was found. Despite the lack of a significant difference between conditions in this contrast, the topographic map and the frequency plot (i.e. based on the positive electrode cluster during the movie period) show a similar descriptive pattern as in the task demand contrast during the movie period. That is, higher theta power for the color-naming than the imitation task with a distribution over mostly left-lateralized over fronto-temporal electrode sites. The lack of significant differences is likely due to a lack in power for this particular contrast.

Supplementary Figure 1.


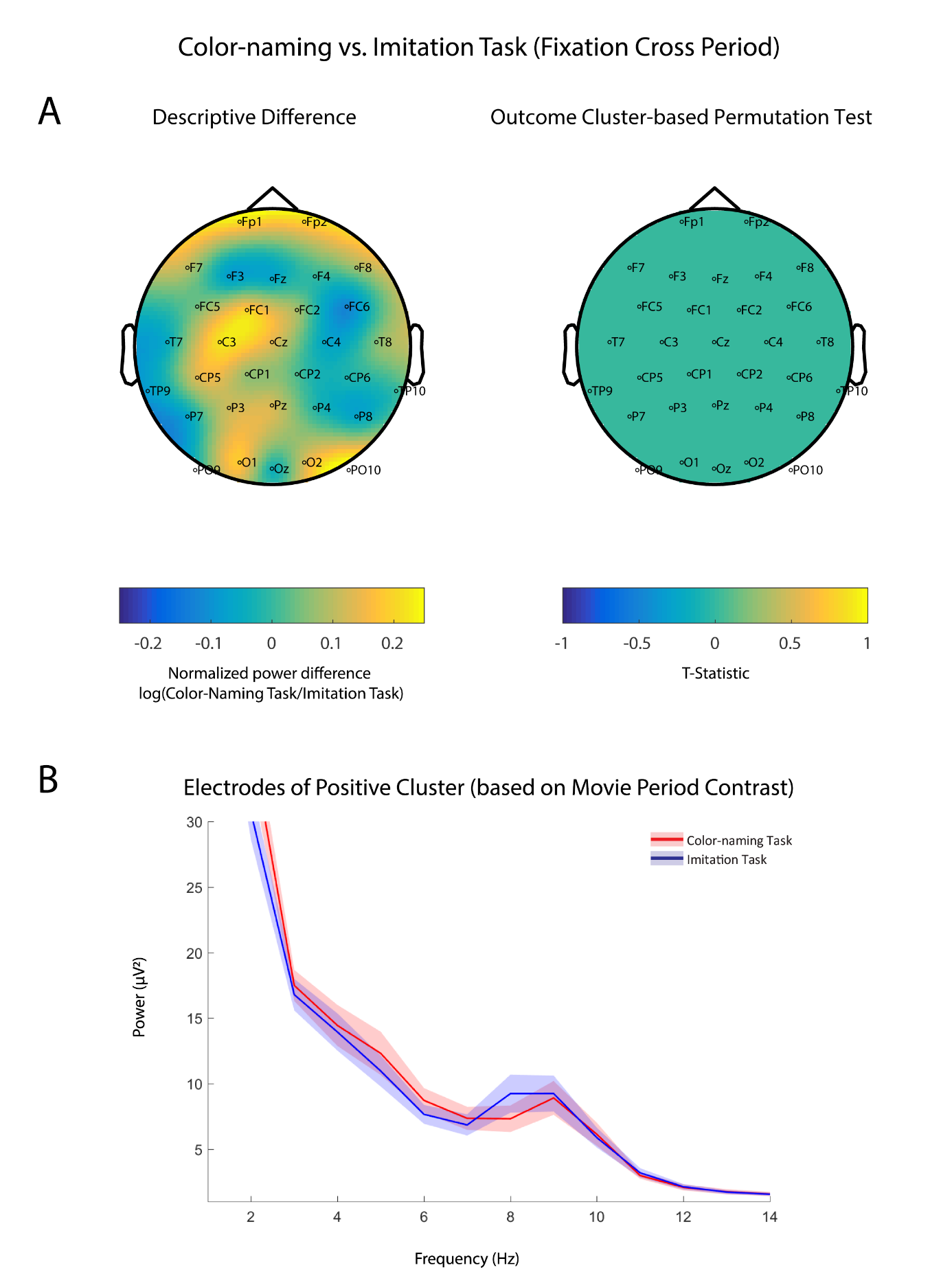


*Supplementary Figure 1.* Panel A (left) illustrates the topography of the descriptive normalized power difference between the Color-Naming and Imitation Task conditions. Warmer colors on the spectrum (reflecting the log-transformed difference values in power) suggest that the difference was most pronounced at the fronto-temporal electrode sites. This is in line with the task demands contrast during the movie period (see Figure 3A). Panel A (right) illustrates results as determined by the cluster-based permutation test contrasting the Color-Naming and Imitation Task conditions The overall green color indicates no positive clusters were found in the test. Note that this comparison is based on the fixation cross period. Panel B displays power values as a function of frequency (Hz) separately for the Color-Naming (red) and Imitation Task (blue) condition. Power values were averaged across electrode sites of the positive cluster found in the task demand contrast during the movie period (Fz, FC1, FC2, FC6, T7, C3, CP5, CP1, P7, P3). Shaded areas represent the standard error of power values.

***Additional analysis: Alpha band (7-12Hz)***

For this additional analysis on the alpha frequency band, we used the same approach as for the theta analysis. More specifically, first average power of the alpha band (sample-specific: 7-12Hz) was estimated using multitaper methods. Then cluster-based permutation tests were conducted on all electrodes for the task engagement (Task vs. NoTask) and task demand (Color-Naming vs. Imitation Task) contrasts. Any clusters are reported and followed up with a descriptive frequency plot based on these clusters with a full 1Hz resolution. Frequency plots are displayed for purely descriptive purposes and are not used in any statistical testing.

**Alpha power during task engagement**

The task engagement contrast reveals a positive cluster centered around a set of fronto-central electrodes. As for theta, more alpha power in these electrodes is detected for being engaged in a task than when not having a task. However, the cluster of electrodes that shows this pattern is partly distinct between theta (F3, Fz, F4, FC1, C3, CP1) and alpha (F4, FC1,FC2, Cz) frequency bands. While taking into account the limited spatial resolution of EEG, the topographic distribution suggests a more centred positive cluster for alpha oscillations in contrast to a more frontal cluster evolving along the midline for theta oscillations.

**Alpha power during different task demands**

The task demand contrast for alpha reveals a positive cluster over parietal-occipital electrode sites (Oz, Pz, P4, CP6, PO10). In other words, there is more alpha power when watching a movie while having the task to later name the color of the dispalyed object than when having the task to later imitate the observed action. While the topographic distribution and the involvement of alpha suggests a visual process at play it is somewhat surprising that there is more power for a task with higher demands on processing a visual feature (i.e. color). The frequency plot of this cluster suggests that this cluster is not showing a very specific effect of alpha power but rather broadband power throughout different frequencies for the color-naming than imitation task. Irrespectively, the task demand contrast results in a cluster distinct from the findings for theta oscillations in this contrast.

Together, outcomes of both contrasts, task engagement and demands, appear somewhat distinct between alpha and theta band oscillations.

Supplementary Figure 2.


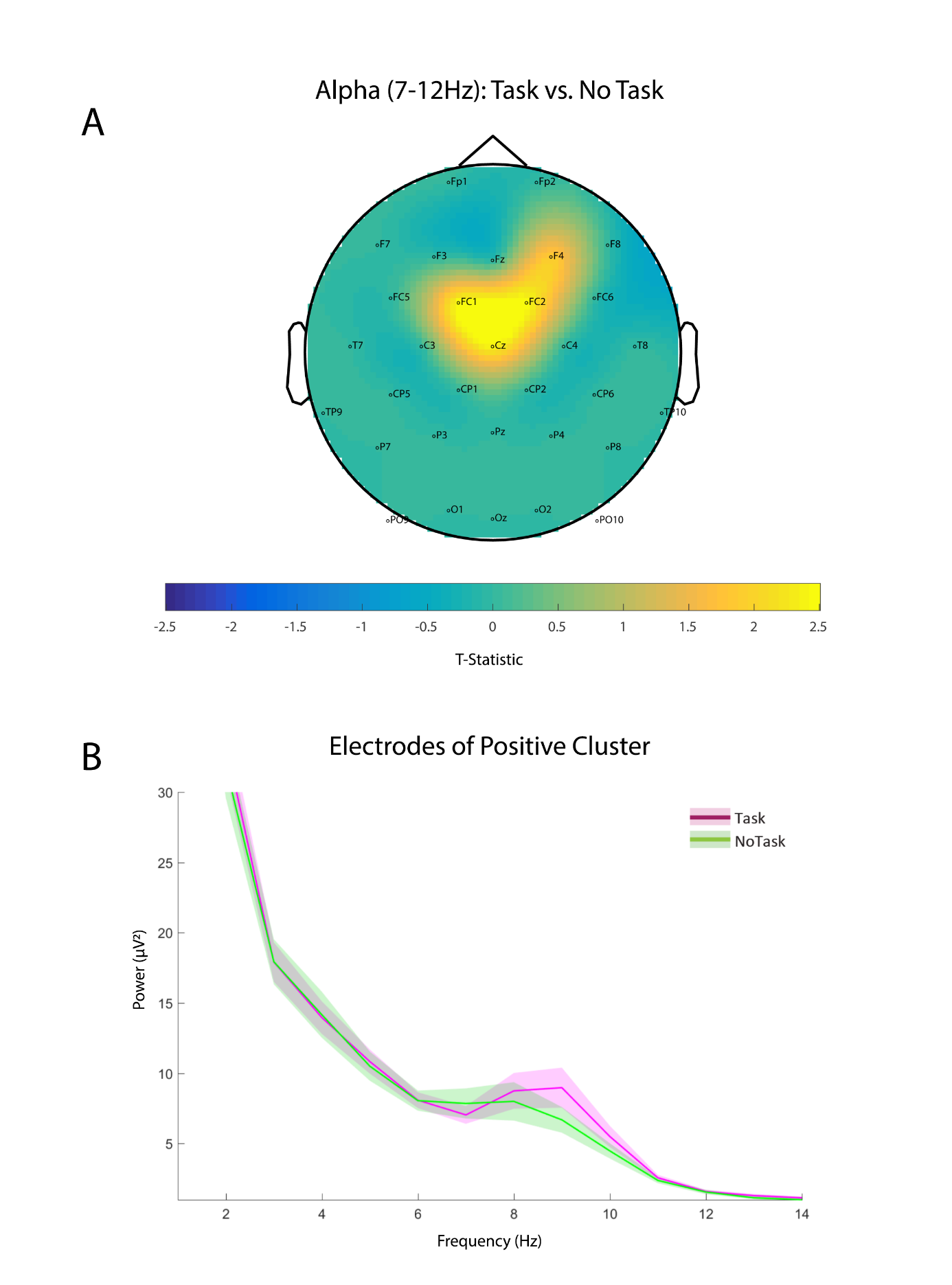


*Supplementary Figure 2.* Panel A illustrates the topography of the results as determined by the cluster-based permutation test contrasting alpha power for the Task and No Task conditions. As indicated by the warmer colors on the spectrum (reflecting the t statistic, with critical t values at +/-2.052), the difference was most pronounced at the fronto-central electrode sites. That is, alpha power (7-12Hz) measured at these sites (F4, FC1, FC2, Cz) was higher in the Task than in the No Task condition. Note that this comparison is based on the fixation cross period. Panel B displays power values as a function of frequency (Hz) separately for the Task (pink) and No Task (green) condition. Power values were averaged across electrode sites of the positive cluster (F4, FC1, FC2, Cz). Shaded areas represent the standard error of power values.

Supplementary Figure 3.
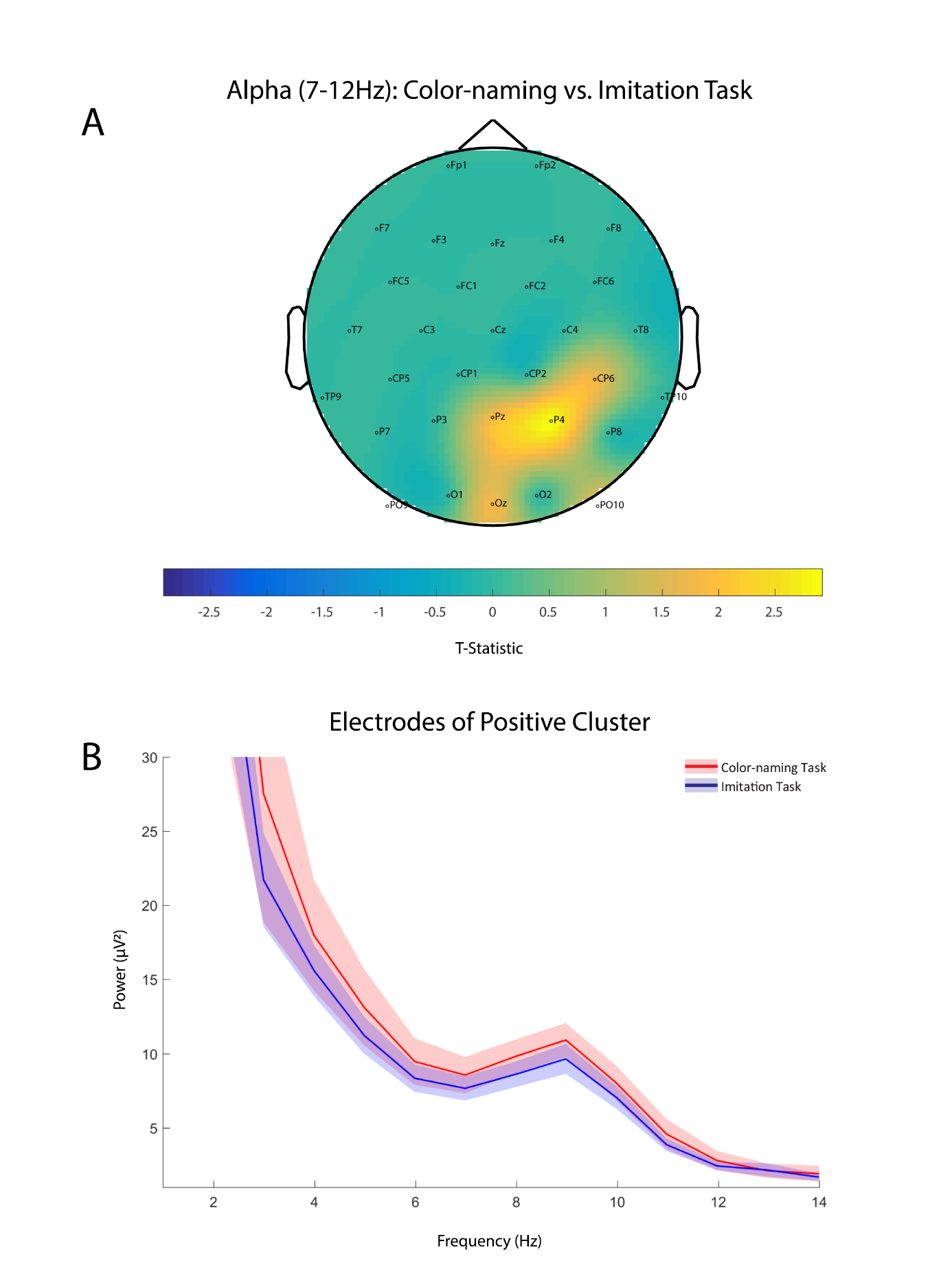


*Supplementary Figure 3.* Panel A illustrates the topography of the alpha results as determined by the cluster-based permutation test contrasting the Color-naming and Imitation Task conditions. As indicated by the warmer colors on the spectrum (reflecting the t statistic, with critical t values at +/-2.052), the difference was most pronounced at the occipital and parietal electrode sites. That is, alpha power (7-12Hz) measured at these sites (Oz, Pz, P4, CP6, PO10) was significantly higher in the Color-naming than in the Imitation Task condition. Note that this comparison is based on the action clip period. Panel B displays power values as a function of frequency (Hz) separately for the Color-naming (red) and Imitation Task (blue) condition. Power values are averaged across electrode sites of the positive cluster (Oz, Pz, P4, CP6, PO10). Shaded areas represent the standard error of power values.
